# Refractory Epilepsy-MRI, EEG and CT scan, a Correlative Clinical Study

**DOI:** 10.3889/oamjms.2016.029

**Published:** 2016-02-16

**Authors:** Dijana Nikodijevic, Natalija Baneva–Dolnenec, Dragana Petrovska-Cvetkovska, Daniela Caparoska

**Affiliations:** 1 *University Clinic of Neurology, Medical Faculty, Ss Cyril and Methodius University of Skopje, Skopje, Republic of Macedonia*; 2 *Medical Faculty, Ss Cyril and Methodius University of Skopje, Skopje, Republic of Macedonia*

**Keywords:** refractory epilepsy, neurophysiology methods, neuroimaging methods, diagnosis

## Abstract

**OBJECTIVES:**

Refractory epilepsies (RE), as well as, the surgically correctable syndromes, are of great interest, since they affect the very young population of children and adolescents. The early diagnosis and treatment are very important in preventing the psychosocial disability. Therefore MRI and EEG are highly sensitive methods in the diagnosis and localization of epileptogenic focus, but also in pre-surgical evaluation of these patients. The aim of our study is to correlate the imaging findings of EEG, MRI and CT scan in refractory symptomatic epilepsies, and to determine their specificity in detecting the epileptogenic focus.

**METHODS:**

The study was prospective with duration of over two years, open-labelled, and involved a group of 37 patients that had been evaluated and diagnosed as refractory epilepsy patients. In the evaluation the type and frequency of seizures were considered, together with the etiologic factors and their association, and finally the risk for developing refractory epilepsy was weighted. EEG and MRI findings and CT scan results were evaluated for their specificity and sensitivity in detecting the epileptogenic focus, and the correlation between them was analyzed.

**RESULTS:**

Regarding the type of seizures considered in our study, the patients with PCS (partial complex seizures) dominated, as opposed to those with generalized seizures (GS) (D=1.178, p < 0.05). Positive MRI findings were registered in 28 patients (75.7%). Most of them were patients with hippocampal sclerosis, 12 (42.8%), and also they were found to have the highest risk of developing refractory epilepsy (RE) (Odds ratio = 5.7), and the highest association between the etiologic factor and refractory epilepsy (p < 0.01). In detecting the epileptogenic focus, a significant difference was found (p < 0.01) between MRI and CT scan findings, especially in patients with hippocampal sclerosis and cerebral malformations. There was a strong correlation between the MRI findings and the etiologic factor (R = 1), and for CT scan and etiologic factor an R=0.75 correlation. There was a significant difference between imaging methods MRI/CT (p < 0.1), and CT/EEG (p < 0.05) in detecting the etiologic factor, and little difference was noticed between findings of EEG/MRI.

**CONCLUSION:**

Our study confirms that for an accurate diagnosis of refractory epilepsy in patients, a combination of neuroimaging and neurophysiologic methods is required. MRI showed to be highly sensitive in detecting the etiologic factor in RE patients, whereas EEG was sensitive in localization of the epileptogenic focus, with high correlation between these two methods. An early diagnosis of these patients is very important in having a better therapeutic response and prognosis for them.

## Introduction

Refractory epilepsies are a specific group that pose a great challenge for diagnosis and treatment to physicians because of their low therapeutic benefit with all the AED [1]. The distribution of these is more often noticed in patients with detectable structural lesion, cerebral tumors, infections, vascular malformations, and hippocampal sclerosis. This group of patients is particularly important because they are under risk of psychosocial disability, irreversible and/or prolonged illness, and pose a serious therapeutic problem. Surgically correctable syndromes are a group of intractable epilepsies, where early surgical treatment reassures reduction of seizure frequency, and prevention of disability [2, 3]. Hippocampal sclerosis as a prototype of these is detected in 40-70% of surgically correctable syndromes [4]. Others, such as small structural lesions, glial tumors, congenital malformations, or anomalies in migration are less often detected. Magnetic resonance imaging is a mandatory method in defining the syndrome in these epilepsies, precise localization of epileptogenin focus, and pre-surgical evaluation. MRI enhances the detection of low grade gliomas, cavernomas, focal cortical displasia, and disembrioplastic tumors [5]. Compared to CT scan, MRI has 80%-100% sensitivity in detecting this structural lesion, whereas the CT scans only 1%. SPECT and PET scan also take a pertinent place in pre-surgical evaluation of these patients.

Other studies have shown however that the CT scan has specificity and sensitivity in detecting the epileptogenic macrofactor in about 60% of cases. Conventional EEG and video EEG are still the main neurophysiologic methods in detecting epileptic abnormalities, diagnosis of the type of epilepsy, and determining the epileptic focus. Besides medicament treatment, surgical treatment of refractory epilepsy can be of great help, especially in cases of surgically correctable syndromes. The interventions that are most commonly used are partial lobectomies. The outcome and prognosis in patients with hippocampal sclerosis, DNT, cavernomas, is very good. In that sense 59% of the patients have good remission after surgery, 19% have reduction of seizures, and 2% remain unchanged. The early identification of refractory epilepsies, structural lesions and epileptogenic focus are crucial for their successful surgical treatment and further prognosis.

The aim of the study is to determine the specificity and sensitivity of neurophysiologic and neuroimaging methods, in detecting the epileptogenic focus of patients with refractory epilepsy, as well as to analyze the correlation between them.

## Material and Methods

The study was prospective, open-labeled, with duration of two-years, and involved 37 patients previously diagnosed as refractory epilepsies. Patients were classified by age, type of seizures, and frequency was determined with the Bohen seizure frequency scoring system.

Nevertheless, the type of seizures, underlying structural lesions as etiologic factors, frequency of seizures, as well as correlation and association of the type of seizures and etiologic factors were evaluated. A conventional EEG was performed by standard 10-20 SI, and underlying epileptiform abnormalities were specified. The MRI was performed with the use of standard sequences, sagital, axial and coronal, T1 and T2, including FLAIR. In the CT scan standard techniques and sequences (16-18 transaxial sequences) were used, and contrast was used where required. Also where there was a certain need, coronal and sagital reconstructions were made. The findings of MRI, EEG and CT were evaluated in detecting various etiologic factors, as well as the correlation with the epileptogenic lesion. The sensitivity and specificity of these methods in detecting the epileptogenic focus was determined.

The statistical program STATISTICA for Windows was used for data elaboration. For testing the significance of difference among specific parametars, the Fisher exact test was used, Kolmogorov Smornov test, and Man Whitney U test. Odds ratio was used to examine risk for certain etiologic factors. Spearman coefficient of correlation was used to determine the relationship between some parameters. Values of p < 0.05 were considered as significant and 0.01 as highly significant.

## Results

A group of 37 patients diagnosed as refractory epilepsy at the age 2-57 (14 male, 23 female) were included in the study. The evaluated frequency of seizures showed that 24 patients had weekly seizures and 13 were with daily seizures.

The distribution of the type of seizures was evaluated, and 23 patients were found to have partial complex seizures PCS (62.1%), whereas 9 (24.3%) had generalized seizures, and simple partial seizures were registered in 5 patients (13.5%). The presence of PCS is dominant and significant as for D = 0.178 and p < 0.05 (Table 1).

**Table 1:** Patients with refractory epilepsy and the type of seizures

Type of seizures	Patients (No)	Patients (%)
Partial simple seizures (PSS)	5	13.5
Partial complex seizures (PCS)	23	62.1
Generalized seizures (GS)	9	24.3

Positive (abnormal) MRI findings were registered in 28 patients (75.7%) (Table 2).

**Table 2:** MRI findings in patients with refractory epilepsy

MRI findings	Patients (No)	Patients (%)
Normal findings	9	24.3
Pathological findings	28	75.7

The highest Bohen score was found in patients with hippocampal sclerosis (102), cerebral malformations (44) and postcerebrovascular illness (26) (Table 3).

**Table 3:** Frequency of seizures in different etiological factors

Etiological factor	Patients No	Frequency of seizures % (weekly)	Frequency of seizures % (daily)	Score Bohen
Cerebral malformation	5	40	60	44
Post cerebrovascular accident	3	33,3	66.6	26
Vascular malformation	2	100		16
Perinatal trauma	1	100		8
Tumors	2		100	18
Hippocampal sclerosis	12	50	50	102
Posttraumatic	2	100		16
Postinflamation, demielinisation	1	100		8
Total	28	53.5	46.4	

The Table 4 shows distribution of different etiological factors. Twenty eight patients had pathological findings, in which those with hippocampal sclerosis 12 (42.8%) and cerebral malformations 5 (17.8%) dominated, whereas traumatic lesions, vascular malformations and tumors were found in 6 patients. Hippocampal sclerosis and cerebral malformations dominated with 60.7% of all patients with cerebral lesions. The assessment of association of the etiologic factor and RE, showed that in all factors except in hippocampal sclerosis there was no significant association, whereas the association of hippocampal sclerosis patients was significant for p < 0.01, Odds ratio = 5.7, showing the risk for refractory epilepsy in patients with hippocampal sclerosis.

**Table 4:** Type of etiological factors in patients with RE

Etiological factor	Patients No	Patients %	X²	Fisher	p	Sig./n.sig.	Odds ratio
Trauma	2	7.14		*	p>0.05	n.sig.	0.31
Cerebral malformation	5	17.8		*	p>0.05	n.sig.	1.0
Post cerebrovascular accident	3	10.7		*	p>0.05	n.sig.	0.97
Vascular malformation	2	7.14		*	p>0.05	n.sig.	1.76
Perinatal trauma	1	3.5		*	p>0.05	n.sig.	
Tumors	2	7.14		*	p>0.05	n.sig.	1.15
Hippocampal sclerosis	12	42.8	18.29	102	p<0.01	sig.	5.76
Toxical							0.32
post infection	1	3.5		8	p>0.05	n.sig.	0.47
Total	28	100					

The comparison of positive etiologic findings on CT and MRI was shown in Table 5. MRI had positive findings in all 28 patients with positive etiology (100%), and the CT scan was positive in 11 patients (39.2%). A significant difference between these two methods, p < 0.01 was detected. The difference of MRI and CT findings versus various etiologic factors has shown considerable difference in hippocampal sclerosis patients (p < 0.01), and cerebral malformation (p < 0.05), where in other etiologies there was no significant difference between the two imaging methods (p > 0.05). The highest correlation found was the one between MRI findings and the etiologic factor (R = 1), and the correlation of CT scan findings and the etiologic factor (R = 0.75).

**Table 5:** CT scan and MRI findings in patients with RE

Etiological factor	Patients No	Patients with positive MRI No %		Patients with positive CT No %	
Trauma	2	2	7.14	1	3.5
Cerebral malformation	2	2	7.14	1	3.5
Post cerebrovascular accident	3	3	10.7	3	10.7
Perinatal trauma	1	1	3.5	1	3.5
Tumors	2	2	7.14	1	3.5
Hippocampal sclerosis	12	12	42.8	3	10.7
Anomal.migrat.	5	5	17.8	1	3.5
Post infection	1	1	3.5	0	0
Total	28	28	100	11	39.2

The distribution of diagnostic evaluation with MRI, CT and EEG has been shown in Table 6. There is significant difference between the results of MRI/CT (p < 0.01) and for CT/EEG (p < 0.05). No significant difference was registered between the results of MRI and EEG, p > 0.05. Positive findings on MRI were found in 75.6% from all 37 patients included in our study; positive results on CT were registered in 29.7% of the patients and positive findings on EEG were registered in 70.2% of the patients.

**Table 6:** MRI, CT and EEG findings in patients with RE

Patients n=37					

Positive MRI (%)	Positive CT (%)	Positive EEG (%)	MRI/CT	MRI/EEG	CT/EEG
75.6	29.7	70.2	p<0.01	p>0.05	p<0.05

MRI, CT and EEG findings in patients with hippocampal sclerosis and cerebral malformations are shown in Table 7. The results from imaging methods in these two etiologies showed significant difference between the findings of MRI/CT (p < 0.01), CT/EEG p < 0.05, and no significant difference was found between the MRI/EEG results (p > 0.05).

**Table 7:** MRI, CT and EEG findings in patients with hippocampal sclerosis and cerebral malformation

Patients n=17					

Positive MRI (%)	Positive CT (%)	Positive EEG (%)	MRI/CT	MRI/EEG	CT/EEG
100	23.5	70.5	p<0.01	p>0.05	p<0.05

## Discussion

Our study as well as similar ones of other clinical studies confirms MRI to be highly sensitive and a specific method in detection of cerebral structural abnormalities (micro and macroetiolocical factors) in RE patients. It is especially important for diagnosis of those etiologic factors where CT does not give good results such as hippocampal sclerosis, cortical dispasia and low grade tumors. Results from analyzed types of seizures in RE patients showed highest presence of PCS in 62.1%, and the evaluated frequency showed more patients to have weekly seizures (68.8%) versus those with daily seizures (35.2%). The highest frequency of seizures was registered in patients with hippocampal sclerosis, cerebral malformations and tumors. Hence, the resistance to therapy may be probably the result of poor diagnosis, low therapeutic response or the epileptogenicity of the etiologic factor. In finding the etiologic factor, MRI and CT scan showed significant difference. Hippocampal sclerosis (42.8%) and cerebral malformations (17.8%) were with highest incidence in RE patients. The risk for PCS is 5 times higher in patients with hippocampal sclerosis. Highest difference in MRI and CT findings was in patients with hippocampal sclerosis and cerebral malformations [6, 7]. Maximal correlation of MRI and etiologic factor was found as for R=1. In the EEG epileptiform abnormalities were found in 70.2% of patients. The evaluation of correlation/difference between imaging methods and EEG showed significant correlation between MRI/EEG, and difference of CT/MRI results also of the EEG/CT results. The imaging techniques confirm with high sensitivity the anatomic lesion in RE patients, and are the first diagnostic step, but not always do they determine the epileptogenic foci. Thus a clinical and electrophysiological correlation should also be done.

Other studies have shown that EEG and intracranial EEG in the pre-surgical evaluation of RE patients, detect the epileptogenic focus with sensitivity of 60-96% specially in temporal epilepsy, and they correlate positive with the clinical semiology and imaging techniques in 73% of cases [8].

In conclusion, the diagnosis of refractory epilepsies requires a correlation of neurophysiologic and neuroimaging techniques. Having in mind the fact that there is a significant difference in the sensitivity of CT and MRI in diagnosis of various etiologic factors, correlation with EEG is important for the diagnosis and classification of RE and localization of the epileptogenic focus. This will furthermore impact the prognosis of these patients, by and large.
